# The effect of a breastfeeding support programme on breastfeeding duration and exclusivity: a quasi-experiment

**DOI:** 10.1186/s12889-019-7331-y

**Published:** 2019-07-24

**Authors:** S. A. van Dellen, B. Wisse, M. P. Mobach, A. Dijkstra

**Affiliations:** 1 Department of Psychology, University of Groningen, Grote Kruisstraat 2/1, 9712 TS Groningen, The Netherlands; 2 Hanze University of Applied Sciences, Zernikeplein 7, P.O. Box 70030, 9704 AA Groningen, The Netherlands; 3 Durham University Business School, Millhill Lane, Durham, DH1 3LB UK; 4 The Hague University of Applied Sciences, Zernikeplein 7, P.O. Box 70030, 9704 AA Groningen, The Netherlands

**Keywords:** Breastfeeding, Lactation consultant, Breastfeeding duration, Quasi-experimental, Evidence-based practice

## Abstract

**Background:**

Breastfeeding has important positive long-term health consequences for infants and mothers. The World Health Organization recommends that all infants should be exclusively breastfed for six months or longer, and advises continuation of breastfeeding for two years or beyond. However, these recommendations are not met in many countries. This study examined whether a comprehensive, evidence-based breastfeeding intervention, the Breastfeeding Support Programme (BSP), promotes prolonged duration and exclusivity of breastfeeding among its participants.

**Methods:**

A quasi-experimental design was used to compare breastfeeding duration and exclusivity in the BSP group (*N* = 66) to breastfeeding duration and exclusivity in a control group (*N* = 72). Participants who followed the BSP were provided with 6 consults delivered by a lactation consultant. The consults started during pregnancy and continued up until 10 weeks after delivery. Participants in the control group did not follow the BSP. Pretest and posttest questionnaires were administered through the internet. A Cox proportional hazards regression analysis was used to estimate adjusted hazard ratios (HR) and 95% confidence intervals (CI) for cessation of any and exclusive breastfeeding, while controlling for differences at baseline.

**Results:**

The effect of the BSP on survival rates for any and exclusive breastfeeding were significant while controlling for differences between the two groups at baseline (respectively HR = 0.34, *p* < .001 [95% CI = 0.18–0.61] and HR = 0.46, *p* < .001 [95% CI = 0.29–0.72]). Among mothers in the BSP group there was on average 66% less risk of cessation of any breastfeeding and on average 54% less risk of cessation of exclusive breastfeeding at any point in time compared to those in the control group.

**Conclusions:**

The BSP appears to be an effective means to delay cessation of any and exclusive breastfeeding cessation and therefore to increase breastfeeding duration and exclusivity. This is an important finding, because earlier cessation of breastfeeding than desired is a common problem in many countries. Future research into the effectiveness of the BSP could consider random assignment to conditions and test the effectiveness of the intervention in other populations to investigate further whether wide-scale implementation of this intervention could be useful to promote breastfeeding.

**Electronic supplementary material:**

The online version of this article (10.1186/s12889-019-7331-y) contains supplementary material, which is available to authorized users.

## Background

Due to the development and subsequent commercialization of infant formula in the eighteenth, nineteenth and twentieth centuries, there has been a strong decline in breastfeeding rates worldwide [1, 2]. However, an increasing body of research shows that breastfeeding has important positive long-term health consequences for infants and mothers. A 2016 meta-analysis of research into the health consequences of breastfeeding for mothers and children concluded that infants who are breastfed for longer periods have lower infectious morbidity and mortality, fewer dental malocclusions and higher intelligence than infants who are not breastfed or breastfed for shorter periods [2]. Furthermore, breastfeeding may also protect against sudden infant death syndrome (SIDS), decrease the risk of necrotising enterocolitis (NEC) for premature babies, and protect children against overweight and diabetes later in life [2]. The beneficial effects of breastfeeding for mothers include protection against breast cancer, improved birth spacing, and potential protection against diabetes and ovarian cancer [2]. Moreover, it was estimated that scaling up breastfeeding to near universal levels could prevent approximately 823,000 child deaths and 20,000 deaths due to breast cancer worldwide annually. Breastfeeding therefore plays an important role in public health for mothers and children around the globe ([2]; also see [3–6]). As a consequence increasing the prevalence and duration of breastfeeding are important health goals in most nations. The World Health Organization (WHO) recommends that all infants should be exclusively breastfed (i.e., receive only breast milk and no other food or drink) for the first 6 months of life to achieve optimal growth, development, and health. Infants should receive complementary foods from 6 months, while breastfeeding should be continued for up to 2 years or beyond [7]. However, these recommendations are not met in many countries. Recent research by the WHO found in a sample of 194 nations, that only 40% of children younger than 6 months are breastfed exclusively [8]. Research findings also suggest that many mothers would like to breastfeed for longer, and that approximately 60% of US mothers stopped breastfeeding earlier than they desired [9]. Mothers stopped breastfeeding prematurely mainly because they had concerns about maternal or child health and concerns about the breastfeeding process (e.g., lactation and milk-pumping problems). The researchers concluded that professional support could help to address these challenges and help mothers to attain their breastfeeding goals [9]. Although some women cannot breastfeed for physical or medical reasons [10], many women could benefit from breastfeeding support.

In the Netherlands, breastfeeding rates also leave ample room for improvement. Although Dutch breastfeeding rates have gradually increased in the past decades, and 80% of Dutch mothers initiate breastfeeding, only 39% of Dutch babies are exclusively breastfed at 6 months^1^, according to a recent study [11]. Moreover, many women in the Netherlands report difficulties with breastfeeding and do not maintain the practice for as long as they intended [12]. In the past decades, national campaigns, emphasising the health benefits of breastfeeding, have been developed to extend the duration of breastfeeding [13]. As part of these campaigns, virtually all hospitals and maternity organisations in the Netherlands have received Baby Friendly Hospital Initiative (BFHI) certification [14], as developed by WHO in 1991 [15]. With regard to breastfeeding recommendations, Dutch guidelines initially followed the WHO guidelines, advising to breastfeed exclusively for at least six months. However, in 2011 the Dutch breastfeeding recommendations were adjusted, in an attempt to prevent the development of food allergies in children [16, 17]. Since 2011 it is therefore advised that mothers should breastfeed for at least six months [18], but should start with complementary foods when the baby is between 4 and 6 months old, if possible [19]. Therefore, exclusive breastfeeding until six months is no longer recommended in the Dutch breastfeeding guidelines; also no mention is made of continuation of breastfeeding for two years or beyond. These deviating national guidelines may partly explain why the prevalence of (exclusive) breastfeeding in the Netherlands is relatively low, and why breastfeeding support and education is especially important in the Netherlands.

Systematic reviews and meta-analyses on breastfeeding promotion interventions have shown that breastfeeding education and/or support can effectively increase breastfeeding rates [20–26]. For instance, a recent meta-analysis, including 27 randomized controlled trials (RCT’s) and 36,051 mothers, found that breastfeeding support interventions aiming to increase exclusive breastfeeding for 6 months were indeed effective. A subgroup analysis looking into the effects of different types of interventions found that a BFHI intervention, an intervention combining education and support, a professional provider led intervention, an intervention that has a protocol available for the provider training program, and an intervention that takes place both during the prenatal and postnatal periods, all increased the rate of exclusive breastfeeding for 6 months [26]. Likewise, a recent systematic review, focussing specifically on professional support interventions, found that interventions spanning from pregnancy to the postnatal period were more effective than interventions that took place in a shorter period, and that interventions using various methods of education and support were more effective than interventions concentrating on a single method [22]. Moreover, it was found that during pregnancy, the BFHI as well as teaching combined with support were effective approaches. During the postnatal period effective approaches included home visits, telephone support, and the use of breastfeeding centres combined with peer support [22].

The current study examines the effectiveness of a comprehensive, evidence-based, professional support intervention for breastfeeding that was implemented in the Netherlands: the Breastfeeding Support Program (BSP). The BSP was developed by two International Board Certified Lactation Consultants (IBCLC), based on theoretical findings and practical experiences. The Theory of Planned Behaviour (TPB) [27] constitutes the theoretical framework for the BSP. The TPB states that human behaviour is predicted by three kinds of considerations: a person’s general evaluation of a given behaviour (attitude); a person’s beliefs about how relevant others will view the behaviour in question (subjective norm); and a person’s perceived ease or difficulty in performing the behaviour (perceived behavioural control). The attitude, subjective norm and perceived behavioural control combined lead to the formation of a behavioural intention to display a certain behaviour. As a general rule, the more favourable the attitude and subjective norm, and the higher the perceived control, the stronger the person’s intention to perform the behaviour in question. Finally, intention is assumed to be the immediate antecedent of behaviour. The TPB is a well-known framework for designing behavioural change interventions [28], and several studies have shown that the TPB can be successfully applied to breastfeeding [29–32].The BSP applies the TPB by aiming to influence positively a mother’s attitude towards breastfeeding, the subjective norm and her perceived behavioural control. The BSP is not only based on the TPB, but also integrates the empirical research findings from systematic reviews of support interventions for breastfeeding promotion [22, 26], suggesting that the most effective interventions are usually delivered by well-trained professionals, combine education and support, and are long-term and intensive, spanning both the prenatal and postnatal period. Although evidence suggests that all these separate elements should increase the effectiveness of a breastfeeding intervention [22, 26], studies that investigate their combined effect are still largely lacking.

The research question we will answer in this study is: do the mothers enrolled in the BSP engage in prolonged breastfeeding in terms of duration and exclusivity compared to mothers in a control group? Based on the accumulated research into the effectiveness of breastfeeding promotion interventions [20–26], and on studies showing the successful application of the TPB to breastfeeding [29–32], we hypothesized that the BSP is an effective intervention in principle. A test of this hypothesis further facilitates the elimination of unsound or ineffective practices in favour of those that have better outcomes, and as such this study aims to support the implementation of evidence-based practice.

## Methods

### Design and recruitment

The study had a quasi-experimental design (with one experimental group and one control group) with pre- and posttest. This design is common in studies aiming to establish the effectiveness of health-related interventions and is considered to be of relatively high quality in the hierarchy of quasi-experimental study designs [33]. Notably, a quasi-experiment may be preferable over a true experiment (or Randomized Controlled Trial; RCT) for testing the effectiveness of interventions, when randomisation is considered to be not ethical, expedient, or possible [33–35], or to create unwanted bias (e.g., low compliance, selective attrition, and questionable ecological validity) [36]. For the current study, we opted for a quasi-experimental design because randomisation was impractical (a Dutch health insurance company offered the BSP to their clients at the time of the research; we were able to carefully monitor the effects, but had no possibility to intervene), and moreover randomisation was considered to limit the ecological validity (women usually make a personal choice to participate in a breastfeeding programme or not; limiting personal choice could create unwanted bias in testing the effectiveness of such a programme). Because in a quasi-experiment allocation to conditions is not randomized, treatment and control groups may not be comparable at baseline. That is, selection effects can lead to pre-existing differences between treatment groups, which can pose a threat to internal validity [35]. We therefore thoroughly screened and controlled for a broad range of possibly confounding factors (see control variables in the Measurements Section).

Our experimental group consisted of pregnant women who were planning to breastfeed and who made a personal decision to enrol in the BSP (supported by their health insurance) on the BSP website. These women were recruited for this study through the enrolment form for the BSP, where they were asked to indicate if they were interested in participating in a study on breastfeeding experiences. The control group consisted of an independently recruited cohort of pregnant women with breastfeeding intentions, who were recruited through primary care facilities (obstetrician/general practitioner). At those facilities we made an enrolment form available for women who were planning to breastfeed. On this form the women could indicate whether they were interested in participating in a study on breastfeeding experiences. Thus, although women in the intervention group and the control group were recruited separately, they all were pregnant, they all planned to breastfeed and they all self-enrolled on the basis of the same written information. Recruitment for this study was conducted in the period of March 2013 to December 2014^2^. Final inclusion criteria were (1) being pregnant; (2) planning to breastfeed; (3) having access to the internet; (4) having singleton gestation; (5) non-missing data for breastfeeding duration.

### Procedure

All the women who indicated an interest in participating in the study received an e-mail with further instructions and a link to complete an online pretest questionnaire. Invitations to complete the pretest questionnaire were sent from month 6 of pregnancy, making sure participants had some time to consider their breastfeeding plans. Invitations for the posttest questionnaire were sent from 28 weeks after the due date, thus making sure that at least 26 weeks had passed since delivery (health policy in the Netherlands aims to achieve that delivery is never more than 2 weeks after the due date). Both the pretest and posttest questionnaires emphasized that participation in the study was voluntary, that responses would be treated confidentially, that results would be reported anonymously, and that it was possible to withdraw from the study at any time without penalty. To encourage participation in the study, prizes were raffled among the participants. The pretest and posttest questionnaires were linked with the use of participants’ e-mail addresses. All the participants provided their informed consent. The research was approved by the Ethical Committee of Psychology of the University of Groningen, the Netherlands.

### Intervention

The BSP tries to increase the proportion of mothers who breastfeed exclusively for six months or longer by positively influencing 1) the mother’s attitude towards breastfeeding (by focussing on the positive effects of exclusive breastfeeding for 6 months or longer for mother and child), 2) the subjective norm (by explicitly involving the father and by forming a reliable source of support and positive messages about breastfeeding throughout the programme) and 3) the mother’s perceived behavioural control (by providing information, encouragement and practical support to improve breastfeeding skills). As such, the BSP uses most of the behavioural change techniques proven to be effective in health interventions [37]. The BSP combines both education and support, extends from pregnancy to the postnatal period, and uses a protocolled series of six individual consults delivered by an IBCLC.

The protocol for the six consults within the BSP is as follows. 1) The programme begins with an intake consult at the lactations consultant’s office during pregnancy. This consult incorporates the following topics: medical history and breast check-up, breastfeeding experience, information about breastfeeding effects on mother and child, advice about breast care during pregnancy, information about the breastfeeding process, food, smoking, alcohol and drugs, the provision of written information about breastfeeding, and the opportunity to discuss questions and problems. 2) The second consult is held during the first week after delivery, either in the hospital or at the family home and focusses on the breastfeeding process as experienced up to that point. 3) The third consult is conducted by telephone on day 14 after delivery to discuss the breastfeeding process. 4) The fourth consult is again conducted by telephone on day 28 after delivery to discuss the breastfeeding process. 5) The fifth consult is held five weeks after delivery at the lactations consultant’s office and consists of a weighing of the baby, discussion of possible problems, breast check-up, and preparation for return to work if applicable. It also provides an opportunity to ask other questions. 6) The sixth and final consult is held 10 weeks after delivery by telephone and focuses on further support for returning to work (if applicable) and other possible questions and issues that mothers may wish to discuss. The number of in-person consults is fixed, but the timing of the consults can be adjusted if necessary (e.g., in case of urgent breastfeeding problems). Moreover, for the duration of the BSP, participants can always contact their IBCLC by phone for questions. The length of the BSP (until 10 weeks after the baby’s birth) is appropriate because most mothers who discontinue breastfeeding early do so during the first three months, mostly due to lactation problems [27]. Before the start of the program a day-long calibration session was organized for all the participating IBCLCs. The protocol was discussed and an example case was used to agree on its practical application. The fact that all lactation consultants in the BSP were IBCLCs contributed to the consistency of the information.

### Measurements

#### Dependent variables

Two dependent variables were used to assess the effectiveness of the intervention: 1) duration of any breastfeeding and 2) duration of exclusive breastfeeding. We measured these variables by asking three questions in the posttest questionnaire: ‘How many weeks old was your baby when he/she received breast milk for the last time?’ (breast milk was defined in the questionnaire as ‘mother’s milk from the breast or expressed breast milk’), ‘How many weeks old was your baby when he/she received artificial feeding for the first time?’ and ‘How many weeks old was your baby when he/she received solid food for the first time?’

#### Control variables

To screen and control for the comparability of participants in the BSP group and the control group, a total of 45 possible confounders was measured at pretest and at posttest (perinatal variables). We arrived at the list of 45 potential confounders after scrutinizing review articles which focus on the determinants of the duration and exclusivity of breastfeeding [11, 38–41]. These possible confounders included psychosocial variables (attitudes, subjective norms, perceived control, prenatal intention, breastfeeding knowledge, maternal work conditions, social and professional support for breastfeeding and/or artificial feeding), demographic variables (age, level education, relationship status and nationality), and biomedical variables (parity, method of delivery, BMI-index, alcohol usage and smoking). To be exhaustive, maternal or paternal asthma, eczema, hay-fever or other allergies were added to this list. It is explicitly advised in the Netherlands to breastfeed babies at increased risk of these health issues [42, 43], which might result in increased motivation to breastfeed. See Additional file 1 for a complete overview of the 45 possible confounders we measured, including a description of the operationalization.

### Analyses

Comparability between the BSP group and the control group was assessed by comparing both groups on the 45 possible confounding variables by means of univariate analyses. Because the data on breastfeeding duration were censored (some of the mothers were still breastfeeding or breastfeeding exclusively at the time of the post-test) Survival Analysis was most suited for the analysis [44]. Cessation of any and exclusive breastfeeding were taken as the final events for the analysis. The week the infant received breast milk for the last time was considered to be the time to event for cessation of any breastfeeding. The week the infant received artificial feeding or solid food^3^ for the first time was considered to be the time to event for cessation of exclusive breastfeeding. First, Kaplan-Meier plots were used to assess survival for any breastfeeding and exclusive breastfeeding in the BSP and the control group, without controlling for differences between the two groups at baseline. Second, a Cox proportional hazards regression analysis was used to estimate adjusted hazard ratios (HR) and 95% confidence intervals (CI) of breastfeeding cessation, controlling for variables that differed between both groups at baseline. Visual examination of survival plots was carried out to check the proportional hazard assumption [45]. All analyses were performed using the Statistical Package for the Social Sciences (SPSS Version 23). See Additional file 2 for the data set; see Additional file 3, Additional file 4, and Additional file 5 for the analyses performed.

## Results

### Response and attrition

**Fig. 1:**
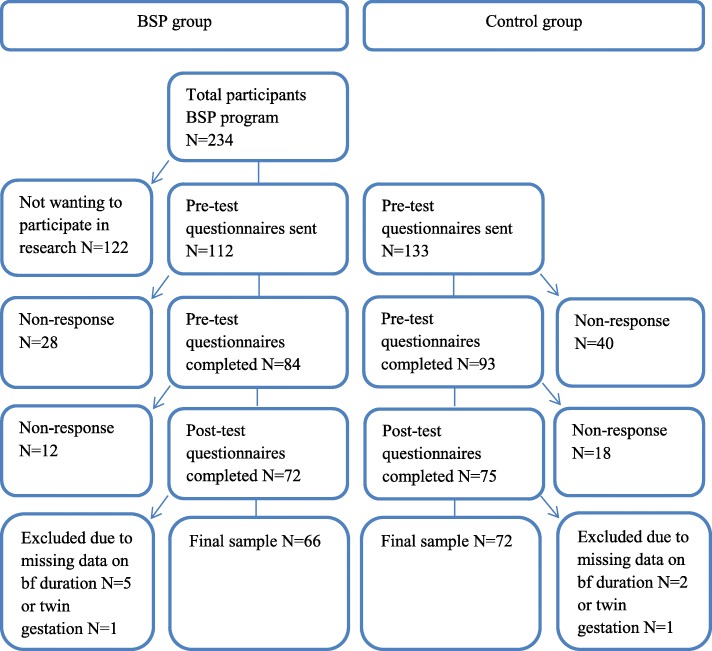
The attrition rates in the BSP group and the control group

A total of 234 women enrolled in the BSP programme (Fig. 1). Of these 234 women, 112 (48%) indicated that they were interested in participating in our study on breastfeeding experiences. In the control group 133 women indicated interest in participation. All of these 245 women received an invitation to participate. We obtained a final sample of 138 women (66 mothers in the BSP group and 72 mothers in the control group), due to non-response at the pretest (*N* = 28 in the BSP group, *N* = 40 in the control group) or posttest (*N* = 12 in the BSP group, *N* = 18 in the control group), missing data on breastfeeding duration (*N* = 5 in the BSP group, *N* = 2 in the control group) or multiple births (*N* = 1 in the BSP group, *N* = 1 in the control group). The response rates in the BSP group and the control group did not differ significantly in the pretest (75.0% versus 69.91%, χ^2^ (1) = 0.781, *p* = .377) or the posttest (58.9% versus 54.1%, χ^2^ (1) = 0.568, *p* = .451).

### Sample description

In the pretest sample 3.5% of the participants reported a lower level education, 22.1% a medium level education and 74.4% a higher level education, and the average age was 31.5 years (*SD* = 4.39). In the posttest sample 1.4% of the participants reported a lower level education, 18.8% a medium level education and 79.7% a higher level education, and their average age was 31.7 years (*SD* = 4.29). The women who dropped out at follow-up had reported mainly lower or medium level education, increasing the proportion of higher educated women in the posttest. Furthermore, the mean age at the time of delivery increased by 0.2 year from pretest to posttest, meaning some of the younger women dropped out. Finally, breastfeeding initiation was almost universal (99.3%) and did not differ significantly between the BSP group and the control group (100% versus 98.6%, χ^2^ (1) = 0.92, *p* = .337).

### Differences between the two groups at baseline

To check for comparability between the BSP group and the control group, the two groups were compared on 45 possibly confounding variables (see Additional file 1) by means of univariate analyses. Eleven differences between the BSP group and the control group were found at baseline with a *p*-value lower or equal to .10 (see Table 1). The mothers in the BSP group experienced more social support for artificial feeding (*p* = .045), had a lower perceived control for breastfeeding (*p* = .039), and would find it more difficult to breastfeed in various situations than the mothers in the control group at baseline (*p* = .062). Furthermore, the mothers in the BSP group were more likely to have been first-time mothers than the mothers in the control group (*p* = .010), had on average less breastfeeding experience (*p* = .003) and had experienced previous breastfeeding less positively than the mothers in the control group at baseline (*p* < .001). The mothers in the BSP group planned to work more hours than the mothers in the control group after their babies were born (*p* = .041). They were also better educated than mothers in the control group (*p* = .002) and the same was true of their partners (*p* = .036). Finally, the mothers in the BSP group were more likely to suffer from asthma (*p* = .011) and were more likely not to have been born in the Netherlands than the mothers in the control group (*p =* .039). A mixed picture emerges: compared to the control group, mothers in the BSP group were mostly characterized by factors which can be expected to have a negative effect on breastfeeding duration and exclusivity (such as experiencing more social support for artificial feeding, having a lower perceived control for breastfeeding, finding it more difficult to breastfeed in various situations, being more likely to be a first-time mother, having less and less positive experiences with previous breastfeeding, planning to work more working hours after the baby is born), but also by some factors which can be expected to have a positive effect on breastfeeding duration and exclusivity (such as being better educated and having better educated partners, a higher asthma incidence and being more likely not to have been born in the Netherlands). The differences between the two groups at baseline were statistically controlled for by including these variables as covariates in the Cox proportional hazards regression analysis.

**Table 1:** Differences between BSP group and control group at baseline (*N* = 138)

	BSP group (*M)*	Control group (*M)*	*p*
Social support artificial feeding (number of people advising artificial feeding)	0.5	0.2	.045
Perceived control breastfeeding (1–5)	3.3	3.6	.039
Expected difficulty breastfeeding in various situations (1–5)	2.9	2.6	.062
First-time mother (%yes)	63.6%	41.7%	.010
Total months of breastfeeding experience	3.2	8.6	.003
Negative experience with previous breastfeeding (1–4)	2.8	1.8	<.001
Anticipated working hours after birth	22.4	18.4	.041
Education level mother			.002
Low	0%	2.8%	
Medium	7.6%	29.2%	
High	92.4%	68.1%	
Education level partner			.036
Low	6.2%	4.2%	
Medium	21.5%	42.3%	
High	72.3%	53.5%	
Asthma mother (% yes)	12.1%	1.4%	.011
Country of origin mother (% not the Netherlands)	9.1%	1.4%	.039

### The effects of the BSP without controlling for differences between groups

**Fig. 2:**
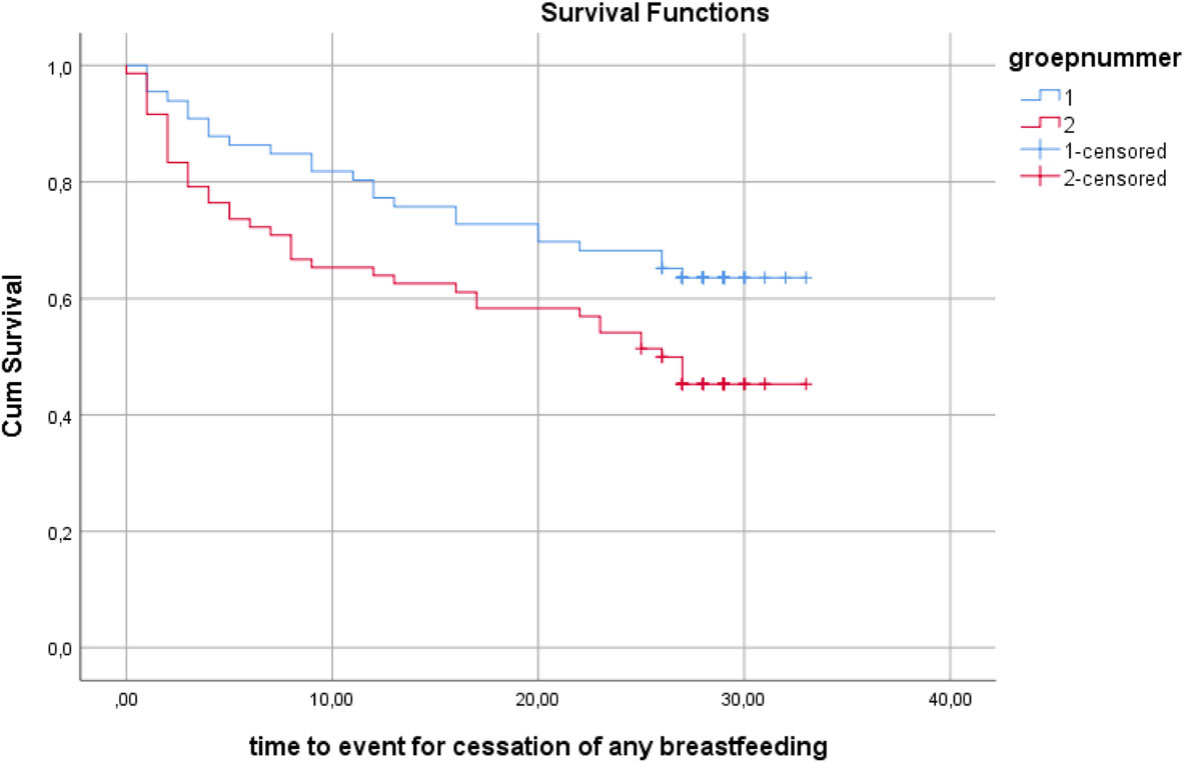
Kaplan-Meier survival estimates for duration of any breastfeeding

**Fig. 3:**
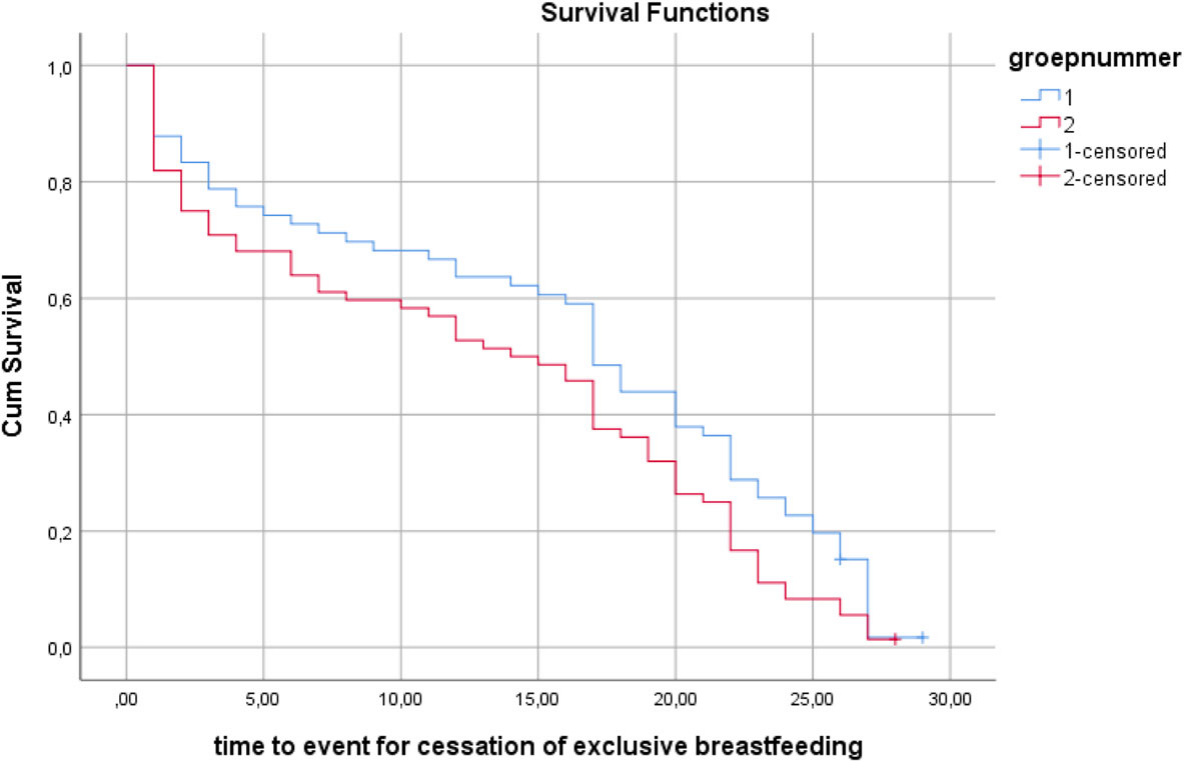
Kaplan-Meier survival estimates for duration of exclusive breastfeeding

First, we used Kaplan-Meier plots to compare the survival curves in the BSP and the control group for duration of any breastfeeding and duration of exclusive breastfeeding, without controlling for differences between the two groups (Figs. 2 and 3). Breastfeeding survival rates were significantly higher in the BSP group than in the control group for any breastfeeding (log-rank test: χ^2^(1) = 4.79, *p* = .029) and for exclusive breastfeeding (log-rank test: χ^2^(1) = 4.07, *p* = .044). The survival curves showed that mothers in the BSP group had a higher probability of breastfeeding and breastfeeding exclusively than mothers in the control group at each point in time. The mean duration of any breastfeeding was 25.08 weeks in the BSP group versus 20.51 weeks in the control group and the mean duration of exclusive breastfeeding was 15.52 weeks in the BSP group versus 12.81 weeks in the control group.

### The effects of the BSP when controlling for differences between groups

A Cox proportional hazards regression analysis was performed, including variables which differed between the two groups at baseline^4^, to correct for potential confounding (see Table 1). The effect of the BSP on survival rates for any breastfeeding was significant while controlling for differences between the two groups at baseline (HR = 0.34, *p* < .001 [95% CI = 0.18–0.61]). The effect of the BSP on survival rates for exclusive breastfeeding was also significant while controlling for differences between the two groups at baseline (HR = 0.46, *p* < .001 [95% CI = 0.29–0.72]). See Table 2. The HRs of 0.34 for any breastfeeding and 0.46 for exclusive breastfeeding indicate that there was on average 66% less risk of cessation of any breastfeeding and on average 54% less risk of cessation of exclusive breastfeeding at any point in time among mothers in the BSP group compared to those in the control group.

**Table 2:** Cox proportional hazards regression analysis for any and exclusive breastfeeding (*N* = 132)

	Any Breastfeeding		Exclusive breastfeeding	
	Hazard ratio	95% CI	Hazard ratio	95% CI
Intervention				
BSP	0.340***	[0.188,0.614]	.458***	[0.291,0.720]
Control	Reference category			
Social support artificial feeding	1.361*	[1.013,1.829]	1.195	[0.931,1.532]
Perceived control breastfeeding (1–5)	0.872	[0.627,1.212]	.741*	[0.585,0.938]
Expected difficulty breastfeeding in various situations (1–5)	1.114	[0.761,1.631]	.935	[0.736,1.189]
First-time mother				
No	5.206***	[2.424,11.183]	1.124	[0.673,1.877]
Yes	Reference category			
Total months of breastfeeding experience	0.854***	[0.784,0.929]	.970*	[0.941,0.999]
How many hours the mother plans to work per week after the baby is born	1.003	[0.979,1.027]	.999	[0.982,1.017]
Education level mother				
Low-medium	.871	[0.421,1.802]	1.094	[0.666,1.798]
High	Reference category		Reference category	
Education level partner				
Low-medium	.936	[0.504,1.737]	1.037	[0.687,1.566]
High	Reference category		Reference category	
Asthma mother				
Yes	.567	[0.130,2.463]	1.443	[0.693,3.005]
No	Reference category		Reference category	
Country of origin mother (% not the Netherlands)				
The Netherlands	.657	[0.228,1.890]	.483	[0.214,1.091]
Not the Netherlands	Reference category		Reference category	

Log Likelihood Test for Any Breastfeeding: χ2(1) = 39.96, *p* < .001

Log Likelihood Test for Exclusive Breastfeeding: χ2(1) = 28.92, *p* = .002

Significance levels: * *p* = < .05, ** *p* = < .01, *** *p* = < .001

### The effects of the BSP among nulliparous women only

To further strengthen the evidence for the effectiveness of the BSP, we attempted to create more comparable groups at baseline by selecting first-time mothers only. This sample of nulliparous women consisted of 72 participants in total (*n* = 42 in the BSP group, and *n* = 30 in the control group). To check for comparability between the BSP group and the control group, the two groups were again compared on 45 possibly confounding variables (see Additional file 1) by means of univariate analyses. Among the nulliparous women, six differences between the BSP group and the control group were found at baseline with a *p*-value lower or equal to .10. The mothers in the BSP group experienced less professional support for breastfeeding (by their obstetrician or course instructor) (*p* = .027), had experienced less stress during the pregnancy (*p* = .069), planned to work more hours after their babies were born (*p* = .017), and planned for a longer pregnancy leave than the mothers in the control group (*p* = .081). Finally, they were better educated than mothers in the control group (*p* = .004) and the same was true of their partners (*p <* .001). First, we used Kaplan-Meier plots to compare the survival curves in the BSP and the control group for duration of any breastfeeding and duration of exclusive breastfeeding, without controlling for differences between the two groups. Breastfeeding survival rates were significantly higher in the BSP group than in the control group for any breastfeeding (log-rank test: χ^2^(1) = 7.85, *p* = .005) and for exclusive breastfeeding (log-rank test: χ^2^(1) = 13.63, *p* < .001). The survival curves showed that mothers in the BSP group had a higher probability of breastfeeding and breastfeeding exclusively than mothers in the control group at each point in time. The mean duration of any breastfeeding was 27.52 weeks in the BSP group versus 19.45 weeks in the control group, and the mean duration of exclusive breastfeeding was 16.76 weeks in the BSP group versus 10.50 weeks in the control group. Finally, a Cox proportional hazards regression analysis was performed, including variables which differed between the two groups at baseline, to correct for potential confounding effects. The results show that the effect of the BSP on survival rates for any breastfeeding was still in the expected direction, but no longer significant (HR = 0.42, *p* = .113 [95% CI = 0.15–1.23]). The effect of the BSP on survival rates for exclusive breastfeeding however was still significant while controlling for differences between the two groups at baseline (HR = 0.35, *p* = .006 [95% CI = 0.17–0.74]). In conclusion, we find similar effects as in our main analysis when assessing the effectiveness of the BSP among nulliparous women only. Although for any breastfeeding the results did not reach significance, the results for exclusive breastfeeding did reach significance, despite a limited sample size.

## Discussion

This study examined whether mothers enrolled in the BSP engage in longer and more exclusive breastfeeding compared to mothers in a control group. Controlling for differences at baseline, there was on average 66% less risk of cessation of any breastfeeding and on average 54% less risk of cessation of exclusive breastfeeding at any point of time among mothers in the BSP group compared to those in the control group. A subgroup analysis, including nulliparous women only, showed similar results, providing evidence for the robustness of the findings. In the current population, the BSP therefore appears to be an effective means to delay cessation of any and exclusive breastfeeding, and therefore to increase breastfeeding duration and exclusivity. This is an important finding, because breastfeeding rates are suboptimal in many countries [8], and interventions which could increase breastfeeding rates are valuable given the positive effects of breastfeeding on the mothers’ and children’s health and well-being [2–6]. Notably, our findings are in line with findings from systematic reviews and meta-analyses showing that breastfeeding promotion interventions can indeed effectively increase breastfeeding rates [20–26].

A strong point of the BSP is that it is a very comprehensive breastfeeding intervention: it combines support and education, is led by a professional provider, has a protocol available, and is implemented during both the prenatal and postnatal periods. The programme is also evidence based, incorporating elements which have been proven to increase the effectiveness of a breastfeeding intervention [22, 26]. Finally, the BSP has a firm theoretical foundation in the Theory of Planned Behaviour [27].

The number of studies evaluating breastfeeding interventions in the Netherlands is very limited: only two other studies are known to us. One study evaluated a breastfeeding intervention aimed at extending the continuation of breastfeeding until at least 3 months by educating postpartum health professionals, but found no significant effect [46]. Another study evaluated an educational programme to promote exclusive breastfeeding for 6 months in families with a history of asthma: breastfeeding exclusively at 6 months was significantly higher in the intervention group than in the control group [47]. In comparison to this last study, the BSP offers the added benefit that it is not tailored to a specific group, but is in principle applicable to the general population. Therefore, the BSP might be deployed as an effective general support measure for mothers intending to breastfeed, to improve the relatively low breastfeeding rates in the Netherlands [11].

An important limitation of the current research is that no randomization was performed, which led to pre-existing differences between groups. Although we believe we had valid reasons to opt for a quasi-experimental design (basing our decision on practical and ecological grounds), the lack of randomization could have resulted in pre-existing differences between the control and the intervention group that affected our findings [33]. Pre-existing differences can pose a threat to internal validity, mainly if they are related to the outcome variable of interest, and can thus provide an alternative explanation for the effect of the intervention. Therefore, the quality of any quasi-experiment is dependent on the degree of comparability between treatment groups, and it is essential to screen and control for possibly confounding factors [33]. In the current study we used post-hoc adjustment to control for potential confounders; another possibility is to prospectively match treatment groups on important confounding variables to create more comparable groups [35]. However, matching can be difficult and sometimes impractical, for example when the sample size is limited compared to the number confounding factors [48], as in the current study. Furthermore, controlling for differences has its limits, in the sense that one cannot control for unmeasured or imperfectly measured confounders [35]. Although we carefully measured and controlled for a broad range of possibly confounding variables in this study, future studies testing the effectiveness of the BSP may consider using alternative designs, most notably those in which participants are randomly assigned to conditions. For example, a RCT where all participants receive some form of BSP, but in different forms or intensities, could prevent selection bias, while at the same time precluding unwanted bias from randomisation (such as low compliance or selective attrition [36]). Studies focussing on the effectiveness of the current intervention at different intensities (e.g., more or fewer consults) and on the effectiveness of its various elements (i.e., which of the elements – information, practical advice or the role of the father etc. – contribute most to the programme’s effectiveness) could also help fine-tune the BSP, potentially making it more effective and efficient.

Another important limitation of the current research (related to the previous point) is that it is unclear to what extent the current findings are generalizable to other populations. The present research focussed explicitly on testing the effectiveness of the BSP among the current participants, and the sample of women in the BSP group was therefore self-selected. As a consequence, it is possible that certain characteristics of the current sample serve as moderators for the effectiveness of the intervention [33]. Two characteristics of our sample seem noteworthy in this respect. First, the women in the BSP group can be characterized by a relatively high education level, and second, it seems that women in the BSP group may have anticipated breastfeeding problems or were planning to return to work. Although we controlled for these differences (making it unlikely that they compromised our results), future research may want to zoom in on their potential effects. For example, the BSP seems to be effective for the women that we investigated, but perhaps it is less effective for, for instance, lower educated women, women who do not anticipate breastfeeding problems, or women who do not plan to return to work. It seems likely that mothers encountering difficulties during breastfeeding could particularly benefit from participating in a BSP. Evidence to this effect could point towards the effectiveness of targeting pregnant women with a higher propensity towards breastfeeding problems. Because the present research showed promising effects in the current population, future research could consider sampling from a broader set of populations to test the differences of BSP effectiveness between sub-groups of women and to test the generalizability of the current findings. Finally, future studies could include more dependent variables, such as whether women sought additional breastfeeding support or the extent to which breastfeeding problems are perceived as effectively handled, to provide greater insight into the effects and working mechanisms of the programme.

## Conclusions

Given the important positive long-term health consequences of breastfeeding for infants and mothers [2–6], knowledge about effective breastfeeding support programmes is highly relevant. This research demonstrated that mothers enrolled in the BSP engage in prolonged breastfeeding in terms of duration and exclusivity compared to mothers in a control group. Therefore, we found empirical support for the BSP being effective in its current form and for the current client group. Future research should test the effectiveness of the intervention in other populations and use randomization to determine whether wide-scale implementation of this intervention could be useful to promote breastfeeding.

## Additional files

Additional file 1: An overview of the 45 possible confounders, including a description of the operationalisation. (DOCX 16 kb)

Additional file 2: Data file BSP anonymized data. (CSV 151 kb)

Additional file 3: Spss syntax for preparation of the data file. (DOCX 16 kb)

Additional file 4: Spss syntax for Cox regression. (DOCX 12 kb)

Additional file 5: Spss syntax for Cox regression nulliparous women only. (DOCX 14 kb)

## Data Availability

All the data generated or analysed during this study are included in this published article and its additional files.

## Abbreviations

BFHI: Baby Friendly Hospital Initiative
BSP: Breastfeeding Support Program
IBCLC: International Board Certified Lactation Consultant
RCT: Randomized controlled trial
TPB: Theory of Planned Behaviour
WHO: World Health Organisation

## Footnotes

1 In this research exclusive breastfeeding was defined as still receiving breast milk, without artificial feeding. No information was recorded in this research on complementary feeding (i.e.: consumption of other foods or drinks alongside breastfeeding or artificial feeding).

2 Since the BSP is a relatively small-scale programme, recruitment was spread out over a longer period to ensure sufficient participants for a reliable effect measurement.

3 Since questions about drinks other than infant formula were not included in our questionnaire, this was not included in our operationalization of exclusive breastfeeding. However, we estimate that it is unlikely that children would have received other drinks than formula, unless they had also begun to receive solid foods and/or formula, since the Dutch Nutrition Centre advises introducing solid foods from month 4-6, and not to introduce water or other drinks until after month 6 [18, 19]. If parents had taken the step not to introduce solid foods or formula before 6 months, then they would most likely not have introduced other drinks either before 6 months. The operationalization used therefore offers a good approximation of exclusive breastfeeding according to the WHO definition.

4 A negative experience with previous breastfeeding is the only variable which differed at baseline but was not controlled for in the Cox proportional hazards regression analyses. We decided not to control for this variable because first-time mothers were unable to answer this question. Note that a negative experience with previous breastfeeding was negatively correlated to breastfeeding duration in the current sample (*r* = -.40, *p* = 0.001). Since the participants in the BSP group scored higher on this variable than participants in the control group, not controlling for this variable leads to a more conservative estimate. The decision not to control for this variable therefore seems justified. Furthermore, because there were almost no individuals with low-level education, we dichotomized education level and education level of the partner into low-medium versus high level to improve reliability of the estimates.
