# Supplementary material for: The effect of a breastfeeding support programme on breastfeeding duration and exclusivity: a quasi-experiment
Source: BMC Public Health. 2019 Jul 24;19:993. doi: 10.1186/s12889-019-7331-y (PMC6657127; doi:10.1186/s12889-019-7331-y)
Supplement: Supplementary file 1 — An overview of the 45 possible confounders, including a description of the operationalisation. (DOCX 16 kb) [file 12889_2019_7331_MOESM1_ESM.docx]

Appendix 1: An overview of the 45 possible confounders, including a description of the operationalisation

| Variable | Description of the operationalisation: |
| --- | --- |
| 1. Attitude breastfeeding | Six items on breastfeeding: unpleasant-pleasant, unhealthy-healthy, not nice-nice, not bothersome-bothersome, unwise-wise, unimportant-important (operationalized according to the guidelines of Ajzen [1]) |
| 1. Attitude artificial feeding | Five items on artificial feeding: unpleasant-pleasant, unhealthy-healthy, not nice-nice, not bothersome-bothersome, unwise-wise (operationalized according to the guidelines of Ajzen [1]) |
| 1. Subjective norm breastfeeding | ‘Most people who are important to me think I definitely should/shouldn’t breastfeed.’ (operationalized according to the guidelines of Ajzen [1]) |
| 1. Subjective norm artificial feeding | ‘Most people who are important to me think I definitely should/shouldn’t use artificial feeding.’ (operationalized according to the guidelines of Ajzen [1]) |
| 1. Social support breastfeeding | The number of the following people who recommended breastfeeding: partner, mother, mother in law, other family members, friends, colleagues (adapted from Gijsbers et al. [2,3]) |
| 1. Social support artificial feeding | The number of the following people who recommended artificial feeding: partner, mother, mother in law, other family members, friends, colleagues (adapted from Gijsbers et al. [2,3]) |
| 1. Professional support breastfeeding | The number of the following people who advised to breastfeed: obstetrician, course instructor (adapted from Gijsbers et al. [2,3]) |
| 1. Professional support artificial feeding | The number of the following people who advised to use artificial feeding: obstetrician, course instructor (adapted from Gijsbers et al. [2,3]) |
| 1. Intention breastfeeding | Two items on breastfeeding: ‘Do you plan to breastfeed during the first six months?’ (definitely not - definitely) ‘Is it likely that you will breastfeed during the first six months?’ (not at all likely – very likely) (operationalized according to the guidelines of Ajzen [1]) |
| 1. Intention artificial feeding | Two items on artificial feeding: ‘Do you plan also to use artificial feeding during the first six months?’ (definitely not - definitely) ‘Is it likely that you will also use artificial feeding during the first six months?’ (not at all likely – very likely) (operationalized according to the guidelines of Ajzen [1]) |
| 1. Perceived control breastfeeding (1-5) | ‘How hard or easy do would you find breastfeeding for six months?’ (very hard – very easy) ‘How much control do you think you have over breastfeeding during six months?’ (no control – total control) ‘If I wanted to breastfeed during six months, I could.’ (I totally disagree - I totally agree) (operationalized according to the guidelines of Ajzen [1]) |
| 1. Expected difficulty breastfeeding in various situations (1-5) | ‘How difficult would it be for you to breastfeed in the following circumstances: in the company of my partner, in the company of other family, in the company of close friends, in the company of acquaintances, in the company of unfamiliar women, in the company of unfamiliar men, in a public space (e.g., a restaurant or train), outside (e.g., in the park)?’ (not at all difficult – very difficult) (adapted from Gijsbers et al. [2,3]) |
| 1. Modelling breastfeeding | ‘How many mothers in your environment have breastfed for 6 months or longer?’ (adapted from Gijsbers et al. [2,3]) |
| 1. Knowledge on breastfeeding [1] | Sum score of 15 multiple-choice questions on breastfeeding (right/wrong/do not know). Item content included increasing milk supply, sore nipples, the law concerning breast-feeding at work and safe milk storage. Possible scores on the test ranged from 0 to 15. (adapted from Gijsbers et al. [2,3]) |
| 1. Planned pregnancy (%yes) | ‘Was this pregnancy planned?’ |
| 1. First-time mother (%yes) | ‘Do you have children already?’ |
| 1. Experience with breastfeeding (%yes of non-first-time mothers) | ‘Have you breastfed these children?’ |
| 1. Total months of breastfeeding experience | ‘How long have you breastfed these children?’ Indicate the number of months per child (child 1 through 5) |
| 1. Negative experience with previous breastfeeding (higher score means more negative) | ‘How did you experience breastfeeding generally with your older children?’ (positively/ more positively than negatively/ not positively, but not negatively either/ more negatively than positively) |
| 1. Stress during pregnancy (1-10) | ‘To what extent did you experience stress during this pregnancy (up until now)?’ (Scale 1-10; very little - very much) |
| 1. Whether the mother plans to work after the baby is born (%yes) | ‘Do you plan to work outdoors after the delivery?’ |
| 1. How many hours the mother plans to work per week after the baby is born | ‘If yes, then how many hours?’ |
| 1. The number of weeks after the baby is born before the mother plans to start working | ‘How many weeks leave do you have after the due date?’ (including vacation and other leave) |
| 1. Age | Age at the time of the due date |
| 1. Partner (yes/no) | ‘Do you have a partner?’ |
| 1. Education level mother | ‘What is the highest level education you completed?’ |
| - 1. Low | elementary school/ lower vocational education/ prevocational education |
| - 1. medium | senior secondary education/ secondary vocational education |
| - 1. high | higher secondary education, higher professional education, science education |
| 1. Education level partner | ‘What is the highest level education your partner completed?’ |
| - 1. low | elementary school/ lower vocational education/ prevocational education |
| - 1. medium | senior secondary education/ secondary vocational education |
| - 1. high | higher secondary education, higher professional education, science education |
| 1. Asthma mother (%yes) | ‘Do you have asthma?’ |
| 1. Eczema mother (%yes) | ‘Do you have eczema?’ |
| 1. Hay fever mother (%yes) | ‘Do you have hay fever?’ |
| 1. Other allergies mother (%yes) | ‘Do you have other allergies?’ |
| 1. Asthma partner (%yes) | ‘Does your partner have asthma?’ |
| 1. Eczema partner (%yes) | ‘Does your partner have eczema?’ |
| 1. Hay fever partner (%yes) | ‘Does your partner have hay fever?’ |
| 1. Other allergies partner (%yes) | ‘Does your partner have other allergies?’ |
| 1. Pre-pregnant BMI | Self-reported pre-pregnant weight/(Self-reported height squared) |
| 1. Smoking during pregnancy (%yes) | ‘Do you smoke, or did you smoke during this pregnancy?’ |
| 1. Alcohol intake during pregnancy (%yes) | ‘Do you use alcohol, or did you use alcohol during this pregnancy?’ |
| 1. Country of origin mother (% not the Netherlands) | ‘What is your country of birth?’ |
| 1. Country of origin partner (% not the Netherlands) | ‘What is your partner’s country of birth?’ |
| 1. Premature birth (%<37 weeks), | 40 weeks -/- (Birth date -/- Due date in weeks) |
| 1. Birth weight | ‘How much did your baby weigh at birth? (in grammes)’ |
| 1. Form of delivery (% Caesarean section) | ‘Did you have a natural birth or a caesarean section?’ |
| 1. Hospital delivery (%yes) | ‘Where did you give birth?’  (home/hospital/somewhere else) |
| 1. Hospital delivery with medical indication (% of hospital deliveries) | ‘Was there a medical indication for your hospital delivery?’ |

**References**

1. Ajzen I. The theory of planned behavior. Organ Behav Hum Decis Process. 1991;50(2):179–211. https://doi.org/10.1016/0749-5978(91)90020-T.
2. Gijsbers B, et al. Factors associated with the initiation of breastfeeding in asthmatic families: the attitude–social influence–self-efficacy model. Breastfeed Med. 2006;1(4):236–46.
3. Gijsbers B, et al. Factors associated with the duration of exclusive breast-feeding in asthmatic families. Health Educ Res. 2007;23(1):158–69﻿.
