# Supplementary material for: The effect of a breastfeeding support programme on breastfeeding duration and exclusivity: a quasi-experiment
Source: BMC Public Health. 2019 Jul 24;19:993. doi: 10.1186/s12889-019-7331-y (PMC6657127; doi:10.1186/s12889-019-7331-y)
Supplement: Supplementary file 3 — Spss syntax for preparation of the data file. (DOCX 16 kb) [file 12889_2019_7331_MOESM3_ESM.docx]

**Spss syntax for preparation of the data file**

* Encoding: UTF-8.

* controle groep en bsp groep in 1 excel bestand gezet en vervolgens voormeting en nameting aan elkaar gekoppeld mbv mailadres.*

*n=181 > 4 pp hadden de voormeting slechts heel beperkt ingevuld, (alleen kennisvragen, deze pp gewist > n=177*

*verder zijn er 2 twin gestation, die moeten er nog uit, maar eerst checken of resultaten gelijk zijn*

RECODE OPLEIDING (1=1) (2=1) (3=1) (4=2) (5=2) (6=3) (7=3) (8=3) INTO opleiding3niv.

VARIABLE LABELS opleiding3niv 'opleiding3niv(LMH)'.

EXECUTE.

RECODE OPLEIDINGPARTN (1=1) (2=1) (3=1) (4=2) (5=2) (6=3) (7=3) (8=3) INTO opleidingp3niv.

VARIABLE LABELS opleidingp3niv 'opleidingp3niv(LMH)'.

EXECUTE.

RECODE OPLEIDING (1=1) (2=1) (3=1) (4=1) (5=1) (6=2) (7=2) (8=2) INTO opleiding2niv.

VARIABLE LABELS opleiding2niv 'opleiding2niv(LMvsH)'.

EXECUTE.

RECODE OPLEIDINGPARTN (1=1) (2=1) (3=1) (4=1) (5=1) (6=2) (7=2) (8=2) INTO opleidingp2niv.

VARIABLE LABELS opleidingp2niv 'opleidingp2niv(LMvsH)'.

EXECUTE.

RECODE kennisv1 kennisv4 kennisv5 kennisv10 kennisv18 kennisv12 (1=0) (2=1) (3=0) INTO kennisv1W

kennisv4W kennisv5W kennisv10W kennisv18W kennisv12W.

EXECUTE.

RECODE kennisv2 kennisv3 kennisv6 kennisv7 kennisv8 kennisv9 kennisv11 kennisv13 kennisv14

kennisv15 kennisv16 kennisv17 (3=0) (1=1) (2=0) INTO kennisv2W kennisv3W kennisv6W kennisv7W

kennisv8W kennisv9W kennisv11W kennisv13W kennisv14W kennisv15W kennisv16W kennisv17W.

EXECUTE.

*vraag 2 11 en 17 zijn omstreden dus laat ik eruit*

COMPUTE kennisBVsomscore=kennisv1W+kennisv4W+kennisv5W+kennisv10W+kennisv18W+kennisv12W+

kennisv3W+kennisv6W+kennisv7W+kennisv8W+kennisv9W+kennisv13W+kennisv14W+

kennisv15W+kennisv16W.

VARIABLE LABELS kennisBVsomscore 'kennisBVsomscore'.

EXECUTE.

COMPUTE intentieBV2items=intentieBV + hoewrschlkBV.

VARIABLE LABELS intentieBV2items 'COMPUTE intentieBV2items=intentieBV + hoewrschlkBV'.

EXECUTE.

*social support: wie heeft je aangeraden bv te geven?*

*social support: wie heeft je aangeraden bv te geven?*

COMPUTE socsupbvtot=SUM(aanrdnBVvk,aanrdnBVpartner,aanrdnBVmoeder,aanrdnBVschmdr,aanrdnBVovfam,

aanrdnBVvrdnnn,aanrdnBVcoll,aanrdnBVcursusl).

VARIABLE LABELS socsupbvtot 'socsupbvtot'.

EXECUTE.

COMPUTE socsupbvprof=SUM(aanrdnBVvk,aanrdnBVcursusl).

VARIABLE LABELS socsupbvprof 'socsupbvprof'.

EXECUTE.

COMPUTE socsupbvsoc=SUM(aanrdnBVpartner, aanrdnBVmoeder,aanrdnBVschmdr,aanrdnBVovfam,aanrdnBVvrdnnn,aanrdnBVcoll).

VARIABLE LABELS socsupbvsoc 'socsupbvsoc'.

EXECUTE.

COMPUTE socsupkvprof=SUM(aanrdnKVvk,aanrdnKVcursusl).

VARIABLE LABELS socsupkvprof 'socsupkvprof'.

EXECUTE.

COMPUTE socsupkvsoc=SUM(aanrdnKVpartner, aanrdnKVmoeder,aanrdnKVschmdr,aanrdnKVovfam,aanrdnKVvrdnnn,aanrdnKVcoll).

VARIABLE LABELS socsupkvsoc 'socsupkvsoc'.

EXECUTE.

COMPUTE EEBV3items=MEAN(EEBV1,EEBV2,EEBV3).

EXECUTE.

RECODE OPVBVVERVELEND OPVKVVERVELEND (1=5) (2=4) (6=3) (7=2) (8=1) INTO ATTBVNIETVERV ATTKVNIETVERV.

EXECUTE.

RECODE OPVBVPLEZIERIG OPVBVGEZOND OPVBVLEUK OPVBVVERSTANDIG OPVBVBELANGRIJK OPVKVPLEZIERIG

OPVKVGEZOND OPVKVLEUK OPVKVVERSTANDIG (1=1) (2=2) (6=3) (7=4) (8=5) INTO ATTBVPLEZ ATTBVGEZOND

ATTBVLEUK ATTBVVERST ATTBVBEL ATTKVPLEZ ATTKVGEZOND ATTKVLEUK ATTKVVERST.

EXECUTE.

COMPUTE ATTBV6i=mean(ATTBVNIETVERV,ATTBVPLEZ,ATTBVGEZOND,ATTBVLEUK,ATTBVVERST,ATTBVBEL).

EXECUTE.

COMPUTE ATTBV5i=mean(ATTBVNIETVERV,ATTBVPLEZ,ATTBVGEZOND,ATTBVLEUK,ATTBVVERST).

EXECUTE .

COMPUTE ATTKV5i=mean(ATTKVNIETVERV,ATTKVPLEZ,ATTKVGEZOND,ATTKVLEUK,ATTKVVERST).

EXECUTE.

RECODE wekenBV (1 thru 25=0) (26 thru Highest=1) INTO zesmaandenBVjanee.

EXECUTE.

DATASET ACTIVATE DataSet1.

RECODE weekeerstKV (Lowest thru 26=0) (ELSE=1) INTO zesmndgeenKVjanee.

VARIABLE LABELS zesmndgeenKVjanee 'zesmndgeenVVjanee'.

EXECUTE.

RECODE weekeerstVV (Lowest thru 26=0) (ELSE=1) INTO zesmndgeenVVjanee.

VARIABLE LABELS zesmndgeenVVjanee 'zesmndgeenVVjanee'.

EXECUTE.

COMPUTE BMI=GEWICHT/(((LENGTE/100))*((LENGTE/100))).

EXECUTE.

RECODE BMI (SYSMIS=SYSMIS) (30 thru Highest=3) (25 thru 30=2) (Lowest thru 25=1) INTO BMI3cat.

EXECUTE.

* Date and Time Wizard: leeftijd.

COMPUTE leeftijd=(bevaldat - gebdatnm) / (365.25 * time.days(1)).

VARIABLE LABEL leeftijd.

VARIABLE LEVEL leeftijd (SCALE).

FORMATS leeftijd (F8.2).

VARIABLE WIDTH leeftijd(8).

EXECUTE.

* leeftijd berekenen, ook voor dropouts, gebaseerd op gebdat vm en uitgedat - NB pp 175 had een verkeerde datum bij uitgdatvm (nl 2013 ipv 2014) *

* En pp 15 en pp 87 hadden bij bevaldatnm en uitgdatnm allebei per ongeluk 2014 ipv 2013 aangegegeven > deze drie aangepast *

COMPUTE leeftijd=(uitgdatvm - gebdatvm) / (365.25 * time.days(1)).

VARIABLE LABEL leeftijd.

VARIABLE LEVEL leeftijd (SCALE).

FORMATS leeftijd (F8.2).

VARIABLE WIDTH leeftijd(8).

EXECUTE.

RECODE gebgewichtkind (2500 thru Highest=2) (Lowest thru 2499=1) INTO gebgewichtking2cat.

EXECUTE.

RECODE leeftijd (Lowest thru 30=1) (30 thru 35=2) (35 thru Highest=3) INTO lleftijd3cat.

VARIABLE LABELS lleftijd3cat 'lleftijd3cat'.

EXECUTE.

RECODE kerenlactkthuis kerenlactkprktk kerenlactktel (1=0) (2=1) (3=2) (4=3) (5=4) (6=5) (7=6) INTO

keerlactthuis keerlactkprktk keerlactktel.

EXECUTE.

COMPUTE contactlactkundige=keerlactthuis+keerlactkprktk+keerlactktel.

EXECUTE.

RECODE MENINGMSTMNSNBV (ELSE=Copy) INTO SOCIALNORMBV.

EXECUTE.

RECODE MENINGMSTMNSNBV (ELSE=Copy) INTO SOCIALNORMBV.

EXECUTE.

COMPUTE intentieBV2items=intentieBV + hoewrschlkBV.

VARIABLE LABELS intentieBV2items 'COMPUTE intentieBV2items=intentieBV + hoewrschlkBV'.

EXECUTE.

COMPUTE intentieKV2items=intentieKV + hoewrschlkKV.

VARIABLE LABELS intentieKV2items 'COMPUTE intentieKV2items=intentieKV + hoewrschlkKV'.

EXECUTE.

RECODE MENINGMSTMNSNKV (ELSE=Copy) INTO SOCIALNORMKV.

EXECUTE.

RECODE MENINGMSTMNSNBV (ELSE=Copy) INTO SOCIALNORMBV.

EXECUTE.

COMPUTE modelling6mndBV=MDRSOMGBVMIN6M.

EXECUTE.

COMPUTE primapari=ALKINDEREN.

EXECUTE.

COMPUTE ervaringBVjanee=ALKINDERENBV.

EXECUTE.

COMPUTE ervaringbvtot=0.

EXECUTE.

COMPUTE ervaringbvtot=SUM(0,HOELANGBVK1,HOELANGBVK2,HOELANGBVK3,HOELANGBVK5).

VARIABLE LABELS ervaringbvtot 'ervbvtot'.

EXECUTE.

DO IF (ALKINDERENBV = 1).

RECODE ervaringbvtot (0=SYSMIS).

END IF.

EXECUTE.

COMPUTE EEBVSITUATIES=MEAN(EEBVSITPARTNER,EEBVSITOVFAM,EEBVSITVRIENDEN,EEBVSITBEKENDEN,

EEBVSITONBEKVR,EEBVSITONBEKMN,EEBVSITOPENBRMT,EEBVSITBUITEN).

EXECUTE.

COMPUTE EBVSITUATIES=(EEBVSITPARTNER+EEBVSITOVFAM+EEBVSITVRIENDEN+EEBVSITBEKENDEN+

EEBVSITONBEKVR+EEBVSITONBEKMN+EEBVSITOPENBRMT+EEBVSITBUITEN) /8.

EXECUTE.

* Date and Time Wizard: wekenanuitgdat.

COMPUTE wekenanuitgdat=(bevaldat - uitgdatnm) / (7 * time.days(1)).

VARIABLE LABELS wekenanuitgdat.

VARIABLE LEVEL wekenanuitgdat (SCALE).

FORMATS wekenanuitgdat (F8.2).

VARIABLE WIDTH wekenanuitgdat(8).

EXECUTE.

COMPUTE gestationalageunder37w=0.

EXECUTE.

COMPUTE gestationalage=40+wekenanuitgdat.

EXECUTE.

IF (gestationalage < 37) gestationalageunder37w=1.

EXECUTE.

COMPUTE socsupkvtot=SUM(aanrdnKVvk,aanrdnKVpartner,aanrdnKVmoeder,aanrdnKVschmdr,aanrdnKVovfam,

aanrdnKVvrdnnn,aanrdnKVcoll,aanrdnKVcursusl).

VARIABLE LABELS socsupkvtot 'socsupkvtot'.

EXECUTE.

COMPUTE socsupkvprof=SUM(aanrdnKVvk,aanrdnKVcursusl).

VARIABLE LABELS socsupkvprof 'socsupkvprof'.

EXECUTE.

COMPUTE socsupkvsoc=SUM(aanrdnKVpartner,aanrdnKVmoeder,aanrdnKVschmdr,aanrdnKVovfam,aanrdnKVvrdnnn,aanrdnKVcoll).

VARIABLE LABELS socsupkvsoc 'socsupkvsoc'.

EXECUTE.

COMPUTE zesmndxclusiefbv=zesmaandenBVjanee * zesmndgeenKVjanee * zesmndgeenVVjanee.

EXECUTE.

*aanvullen van missende waarden die eigenlijk 0 zijn:*

RECODE socsupbvprof (MISSING=0).

EXECUTE.

RECODE socsupbvsoc (MISSING=0).

EXECUTE.

RECODE socsupkvsoc (MISSING=0).

EXECUTE.

RECODE socsupkvprof (MISSING=0).

EXECUTE.

RECODE ervaringbvtot (MISSING=0).

EXECUTE.

DO IF (MISSING(ALKINDERENBV)).

RECODE ervaringbvtot (MISSING=0).

END IF.

EXECUTE.

DO IF (NABEVWERKEN>1).

RECODE NABEVWERKENUREN (MISSING=0).

END IF.

EXECUTE.

RECODE zesmndxclusiefbv (MISSING=0).

EXECUTE.

RECODE zesmaandenBVjanee (MISSING=0).

EXECUTE.

FREQUENCIES VARIABLES=socsupkvsoc primapari ervaringbvtot EEBV3items EEBVSITUATIES NABEVWERKENUREN

opleiding3niv opleidingp3niv ASTMA GEBLANDNLJANEE gestationalageunder37w

/ORDER=ANALYSIS.

RECODE eenofmeerling (MISSING=-999).

EXECUTE.

DO IF (ALKINDERENBV = 1).

RECODE ervaringbvtot (0=SYSMIS).

END IF.

EXECUTE.

* Encoding: UTF-8.

IF weekeerstVV = weekeerstKV wekenexbv = (weekeerstVV -1).

EXECUTE.

IF (MISSING (weekeerstKV)) wekenexbv = (weekeerstVV - 1).

EXECUTE.

IF (MISSING (weekeerstVV)) wekenexbv = (weekeerstKV-1).

EXECUTE.

IF weekeerstKV < weekeerstVV wekenexbv = (weekeerstKV - 1).

EXECUTE.

IF weekeerstVV < weekeerstKV wekenexbv = (weekeerstVV - 1).

EXECUTE.

IF bvjanneniets = 1 wekenexbv = 0.

IF bvjanneniets = 1 wekenbv = 0.

EXECUTE.

* berekenen 4 mnd exclusieve bv, want NL norm is vanaf 4 mnd bijvoeding gaan geven.*

RECODE wekenexbv (SYSMIS=0) (Lowest thru 15=0) (16 thru Highest=1) INTO viermndexbv.

VARIABLE LABELS viermndexbv '4 maanden exlusieve bv'.

EXECUTE.

* berekenen wekeninvulnm*

* Date and Time Wizard: wekeninvulNM.

COMPUTE wekeninvulNM=(StartDatenm - bevaldat) / (7 * time.days(1)).

VARIABLE LABELS wekeninvulNM "aantal weken na bevaldatum waarop namting is ingevuld".

VARIABLE LEVEL wekeninvulNM (SCALE).

FORMATS wekeninvulNM (F8.2).

VARIABLE WIDTH wekeninvulNM(8).

EXECUTE.

* Date and Time Wizard: wekeninvulnmtrunc.

COMPUTE wekeninvulnmtrunc=DATEDIF(StartDatenm, bevaldat, "weeks").

VARIABLE LABELS wekeninvulnmtrunc "weken na belatdatum afgekapt".

VARIABLE LEVEL wekeninvulnmtrunc (SCALE).

FORMATS wekeninvulnmtrunc (F5.0).

VARIABLE WIDTH wekeninvulnmtrunc(5).

EXECUTE.

* event indicator any bf *

IF wekenbv = 28 cessbf = 0.

EXECUTE.

IF wekenbv < 28 cessbf = 1.

EXECUTE.

* time to event any bf *

IF wekenbv < 28 cessbfwk = wekenbv.

EXECUTE.

IF wekenbv = 28 cessbfwk = wekeninvulnmtrunc.

EXECUTE.

* event indicator excl bf *

IF wekenexbv = 27 cessexbf = 0.

EXECUTE.

IF wekenexbv < 27 cessexbf = 1.

EXECUTE.

* time to event excl bf *

IF wekenexbv < 27 cessexbfwk = (wekenexbv + 1).

EXECUTE.

IF wekenexbv = 27 cessexbfwk = wekeninvulnmtrunc.

EXECUTE.

* conservatieve schatting time to event mbt any bf voor pp 44 en pp 8 ivm missing data*

IF ppnumber = 44 cessbfwk = 26.

EXECUTE.

IF ppnumber = 8 cessbfwk = 26.

EXECUTE.
