# Supplementary material for: The effect of a breastfeeding support programme on breastfeeding duration and exclusivity: a quasi-experiment
Source: BMC Public Health. 2019 Jul 24;19:993. doi: 10.1186/s12889-019-7331-y (PMC6657127; doi:10.1186/s12889-019-7331-y)
Supplement: Supplementary file 4 — Spss syntax for Cox regression. (DOCX 12 kb) [file 12889_2019_7331_MOESM4_ESM.docx]

**Spss syntax for Cox regression**

* Encoding: UTF-8.

DATASET ACTIVATE DataSet1.

*main analysis*

COMPUTE eenlingofonbekend=1.

EXECUTE.

IF (eenofmeerling = 2) eenlingofonbekend=0.

EXECUTE.

USE ALL.

COMPUTE filter_$=(eenlingofonbekend =1 & wekenBV >= 0).

VARIABLE LABELS filter_$ 'eenofmeerling < 2 & wekenBV >= 0 (FILTER)'.

VALUE LABELS filter_$ 0 'Not Selected' 1 'Selected'.

FORMATS filter_$ (f1.0).

FILTER BY filter_$.

EXECUTE.

DATASET ACTIVATE DataSet1.

KM cessbfwk BY groepnummer

/STATUS=cessbf(1)

/PRINT TABLE MEAN

/PLOT SURVIVAL

/TEST LOGRANK

/COMPARE OVERALL POOLED.

KM cessexbfwk BY groepnummer

/STATUS=cessexbf(1)

/PRINT TABLE MEAN

/PLOT SURVIVAL

/TEST LOGRANK

/COMPARE OVERALL POOLED.

COXREG cessbfwk

/STATUS=cessbf(1)

/PATTERN BY groepnummer

/CONTRAST (groepnummer)=Indicator

/METHOD=ENTER groepnummer

/PLOT SURVIVAL HAZARDS

/PRINT=CI(95)

/CRITERIA=PIN(.05) POUT(.10) ITERATE(20).

COXREG cessbfwk

/STATUS=cessbf(1)

/PATTERN BY groepnummer

/CONTRAST (groepnummer)=Indicator

/CONTRAST (ASTMA)=Indicator

/CONTRAST (GEBLANDNLJANEE)=Indicator

/CONTRAST (primapari)=Indicator

/CONTRAST (opleiding2niv)=Indicator

/CONTRAST (opleidingp2niv)=Indicator

/METHOD=ENTER groepnummer socsupkvsoc EEBV3items EEBVSITUATIES primapari ervaringbvtot NABEVWERKENUREN

opleiding2niv opleidingp2niv ASTMA GEBLANDNLJANEE

/PLOT SURVIVAL HAZARDS

/PRINT=CI(95)

/CRITERIA=PIN(.05) POUT(.10) ITERATE(20).

COXREG cessexbfwk

/STATUS=cessexbf(1)

/PATTERN BY groepnummer

/CONTRAST (groepnummer)=Indicator

/METHOD=ENTER groepnummer

/PLOT SURVIVAL HAZARDS

/PRINT=CI(95)

/CRITERIA=PIN(.05) POUT(.10) ITERATE(20).

COXREG cessexbfwk

/STATUS=cessexbf(1)

/PATTERN BY groepnummer

/CONTRAST (groepnummer)=Indicator

/CONTRAST (ASTMA)=Indicator

/CONTRAST (GEBLANDNLJANEE)=Indicator

/CONTRAST (primapari)=Indicator

/CONTRAST (opleiding2niv)=Indicator

/CONTRAST (opleidingp2niv)=Indicator

/METHOD=ENTER groepnummer socsupkvsoc EEBV3items EEBVSITUATIES primapari ervaringbvtot NABEVWERKENUREN

opleiding2niv opleidingp2niv ASTMA GEBLANDNLJANEE

/PLOT SURVIVAL HAZARDS

/PRINT=CI(95)

/CRITERIA=PIN(.05) POUT(.10) ITERATE(20).

* checken assumptie van proportionaliteit dmv log minus log plot > parallelle lijnen = ok*

DATASET ACTIVATE DataSet1.

COXREG cessbfwk

/STATUS=cessbf(1)

/STRATA=groepnummer

/PLOT LML

/CRITERIA=PIN(.05) POUT(.10) ITERATE(20).

* cox regression met alleen de significante covariaten*

COXREG cessbfwk

/STATUS=cessbf(1)

/PATTERN BY groepnummer

/CONTRAST (groepnummer)=Indicator

/CONTRAST (primapari)=Indicator

/METHOD=ENTER groepnummer socsupkvsoc primapari ervaringbvtot

/PLOT SURVIVAL HAZARDS

/PRINT=CI(95)

/CRITERIA=PIN(.05) POUT(.10) ITERATE(20).

COXREG cessexbfwk

/STATUS=cessexbf(1)

/PATTERN BY groepnummer

/CONTRAST (groepnummer)=Indicator

/METHOD=ENTER groepnummer EEBV3items ervaringbvtot

/PLOT SURVIVAL HAZARDS

/PRINT=CI(95)

/CRITERIA=PIN(.05) POUT(.10) ITERATE(20).
