# Supplementary material for: The effect of a breastfeeding support programme on breastfeeding duration and exclusivity: a quasi-experiment
Source: BMC Public Health. 2019 Jul 24;19:993. doi: 10.1186/s12889-019-7331-y (PMC6657127; doi:10.1186/s12889-019-7331-y)
Supplement: Supplementary file 5 — Spss syntax for Cox regression nulliparous women only. (DOCX 14 kb) [file 12889_2019_7331_MOESM5_ESM.docx]

**Spss syntax for Cox regression nulliparous women only**

* Encoding: UTF-8.

DATASET ACTIVATE DataSet1.

*main analysis*

USE ALL.

COMPUTE filter_$=(eenlingofonbekend =1 & wekenBV >= 0).

VARIABLE LABELS filter_$ 'eenofmeerling < 2 & wekenBV >= 0 (FILTER)'.

VALUE LABELS filter_$ 0 'Not Selected' 1 'Selected'.

FORMATS filter_$ (f1.0).

FILTER BY filter_$.

EXECUTE.

* filter toegevoegd voor primpipari > primapari= 2 = 1, n=72, 42 in bsp en 30 in controlegroep om na te gaan of de effecten nog steeds hetzelfde zijn*

* NB ik heb gecheckt welke variabelen op baseline verschillen tussen de groepen voor deze selectie en de covariaten daarop aangepast *

COMPUTE eenlingofonbekend=1.

EXECUTE.

IF (eenofmeerling = 2) eenlingofonbekend=0.

EXECUTE.

USE ALL.

COMPUTE filter_$=(eenlingofonbekend =1 & wekenBV >= 0 & primapari =2).

VARIABLE LABELS filter_$ 'eenofmeerling < 2 & wekenBV >= 0 (FILTER)'.

VALUE LABELS filter_$ 0 'Not Selected' 1 'Selected'.

FORMATS filter_$ (f1.0).

FILTER BY filter_$.

EXECUTE.

DATASET ACTIVATE DataSet1.

KM cessbfwk BY groepnummer

/STATUS=cessbf(1)

/PRINT TABLE MEAN

/PLOT SURVIVAL

/TEST LOGRANK

/COMPARE OVERALL POOLED.

KM cessexbfwk BY groepnummer

/STATUS=cessexbf(1)

/PRINT TABLE MEAN

/PLOT SURVIVAL

/TEST LOGRANK

/COMPARE OVERALL POOLED.

* checken voor verschillen op baseline *

* Encoding: UTF-8.

USE ALL.

COMPUTE filter_$=(eenlingofonbekend =1 & wekenBV >= 0 & primapari =2).

VARIABLE LABELS filter_$ 'eenofmeerling < 2 & wekenBV >= 0 (FILTER)'.

VALUE LABELS filter_$ 0 'Not Selected' 1 'Selected'.

FORMATS filter_$ (f1.0).

FILTER BY filter_$.

EXECUTE.

MEANS TABLES=leeftijd socsupkvsoc EEBV3items EEBVSITUATIES ervaringbvtot

NABEVWERKENUREN HOEBVERVAREN BY interventie

/CELLS=MEAN COUNT STDDEV.

UNIANOVA ATTBV6i BY groepnummer

/METHOD=SSTYPE(3)

/INTERCEPT=INCLUDE

/EMMEANS=TABLES(groepnummer)

/CRITERIA=ALPHA(.05)

/DESIGN=groepnummer.

UNIANOVA ATTKV5i BY groepnummer

/METHOD=SSTYPE(3)

/INTERCEPT=INCLUDE

/EMMEANS=TABLES(groepnummer)

/CRITERIA=ALPHA(.05)

/DESIGN=groepnummer.

UNIANOVA SOCIALNORMBV BY groepnummer

/METHOD=SSTYPE(3)

/INTERCEPT=INCLUDE

/EMMEANS=TABLES(groepnummer)

/CRITERIA=ALPHA(.05)

/DESIGN=groepnummer.

UNIANOVA SOCIALNORMKV BY groepnummer

/METHOD=SSTYPE(3)

/INTERCEPT=INCLUDE

/EMMEANS=TABLES(groepnummer)

/CRITERIA=ALPHA(.05)

/DESIGN=groepnummer.

UNIANOVA socsupbvsoc BY groepnummer

/METHOD=SSTYPE(3)

/INTERCEPT=INCLUDE

/EMMEANS=TABLES(groepnummer)

/CRITERIA=ALPHA(.05)

/DESIGN=groepnummer.

UNIANOVA socsupkvsoc BY groepnummer

/METHOD=SSTYPE(3)

/INTERCEPT=INCLUDE

/EMMEANS=TABLES(groepnummer)

/CRITERIA=ALPHA(.05)

/DESIGN=groepnummer.

UNIANOVA socsupbvprof BY groepnummer

/METHOD=SSTYPE(3)

/INTERCEPT=INCLUDE

/EMMEANS=TABLES(groepnummer)

/CRITERIA=ALPHA(.05)

/DESIGN=groepnummer.

UNIANOVA socsupkvprof BY groepnummer

/METHOD=SSTYPE(3)

/INTERCEPT=INCLUDE

/EMMEANS=TABLES(groepnummer)

/CRITERIA=ALPHA(.05)

/DESIGN=groepnummer.

UNIANOVA intentieKV2items BY groepnummer

/METHOD=SSTYPE(3)

/INTERCEPT=INCLUDE

/EMMEANS=TABLES(groepnummer)

/CRITERIA=ALPHA(.05)

/DESIGN=groepnummer.

UNIANOVA intentieBV2items BY groepnummer

/METHOD=SSTYPE(3)

/INTERCEPT=INCLUDE

/EMMEANS=TABLES(groepnummer)

/CRITERIA=ALPHA(.05)

/DESIGN=groepnummer.

UNIANOVA EEBV3items BY groepnummer

/METHOD=SSTYPE(3)

/INTERCEPT=INCLUDE

/EMMEANS=TABLES(groepnummer)

/CRITERIA=ALPHA(.05)

/DESIGN=groepnummer.

UNIANOVA EEBVSITUATIES BY groepnummer

/METHOD=SSTYPE(3)

/INTERCEPT=INCLUDE

/EMMEANS=TABLES(groepnummer)

/CRITERIA=ALPHA(.05)

/DESIGN=groepnummer.

UNIANOVA modelling6mndBV BY groepnummer

/METHOD=SSTYPE(3)

/INTERCEPT=INCLUDE

/EMMEANS=TABLES(groepnummer)

/CRITERIA=ALPHA(.05)

/DESIGN=groepnummer.

UNIANOVA kennisBVsomscore BY groepnummer

/METHOD=SSTYPE(3)

/INTERCEPT=INCLUDE

/EMMEANS=TABLES(groepnummer)

/CRITERIA=ALPHA(.05)

/DESIGN=groepnummer.

UNIANOVA ervaringbvtot BY groepnummer

/METHOD=SSTYPE(3)

/INTERCEPT=INCLUDE

/EMMEANS=TABLES(groepnummer)

/CRITERIA=ALPHA(.05)

/DESIGN=groepnummer.

UNIANOVA leeftijd BY groepnummer

/METHOD=SSTYPE(3)

/INTERCEPT=INCLUDE

/EMMEANS=TABLES(groepnummer)

/CRITERIA=ALPHA(.05)

/DESIGN=groepnummer.

UNIANOVA HOEBVERVAREN BY groepnummer

/METHOD=SSTYPE(3)

/INTERCEPT=INCLUDE

/EMMEANS=TABLES(groepnummer)

/CRITERIA=ALPHA(.05)

/DESIGN=groepnummer.

UNIANOVA STRESSERVARENZW BY groepnummer

/METHOD=SSTYPE(3)

/INTERCEPT=INCLUDE

/EMMEANS=TABLES(groepnummer)

/CRITERIA=ALPHA(.05)

/DESIGN=groepnummer.

UNIANOVA NABEVWERKENUREN BY groepnummer

/METHOD=SSTYPE(3)

/INTERCEPT=INCLUDE

/EMMEANS=TABLES(groepnummer)

/CRITERIA=ALPHA(.05)

/DESIGN=groepnummer.

UNIANOVA WEERWERKNAxWKN BY groepnummer

/METHOD=SSTYPE(3)

/INTERCEPT=INCLUDE

/EMMEANS=TABLES(groepnummer)

/CRITERIA=ALPHA(.05)

/DESIGN=groepnummer.

UNIANOVA leeftijd BY groepnummer

/METHOD=SSTYPE(3)

/INTERCEPT=INCLUDE

/EMMEANS=TABLES(groepnummer)

/CRITERIA=ALPHA(.05)

/DESIGN=groepnummer.

UNIANOVA BMI BY groepnummer

/METHOD=SSTYPE(3)

/INTERCEPT=INCLUDE

/EMMEANS=TABLES(groepnummer)

/CRITERIA=ALPHA(.05)

/DESIGN=groepnummer.

UNIANOVA gebgewichtkind BY groepnummer

/METHOD=SSTYPE(3)

/INTERCEPT=INCLUDE

/EMMEANS=TABLES(groepnummer)

/CRITERIA=ALPHA(.05)

/DESIGN=groepnummer.

CROSSTABS

/TABLES= ZWANGGEPLAND primapari ALKINDERENBV NABEVWERKEN PARTNER opleiding2niv opleidingP2niv ASTMA ECZEEM HOOIKOORTS ANDEREALLERGIE STMAPARTN

ECZEEMPARTN HOOIKOORTSPARTN ANDEREALLERGIEP ROKEN ALCOHOL GEBLANDNLJANEE GEBLANDPARTN gestationalageunder37w

manierbevall plaatsbevall medindicatie eenofmeerling BY groepnummer

/FORMAT=AVALUE TABLES

/STATISTICS=CHISQ

/CELLS=COUNT COLUMN

/COUNT ROUND CELL.

* SORT CASES BY gestationalageunder37w(A). CROSSTABS

/TABLES=primapari ZWANGGEPLAND ALKINDEREN ALKINDERENBV NABEVWERKEN PARTNER opleiding3niv

GEBLANDNLJANEE OPLEIDINGPARTN GEBLANDPARTN ASTMA ECZEEM HOOIKOORTS ANDEREALLERGIE STMAPARTN

ECZEEMPARTN HOOIKOORTSPARTN ANDEREALLERGIEP ROKEN ALCOHOL gestationalageunder37w opleidingP3niv BY

groepnummer

/FORMAT=AVALUE TABLES

/STATISTICS=CHISQ

/CELLS=COUNT COLUMN

/COUNT ROUND CELL.*

* nu de cox regression met de significante verschillen erbij:*

COXREG cessbfwk

/STATUS=cessbf(1)

/PATTERN BY groepnummer

/CONTRAST (groepnummer)=Indicator

/METHOD=ENTER groepnummer

/PLOT SURVIVAL HAZARDS

/PRINT=CI(95)

/CRITERIA=PIN(.05) POUT(.10) ITERATE(20).

COXREG cessbfwk

/STATUS=cessbf(1)

/PATTERN BY groepnummer

/CONTRAST (groepnummer)=Indicator

/CONTRAST (ASTMA)=Indicator

/CONTRAST (GEBLANDNLJANEE)=Indicator

/CONTRAST (primapari)=Indicator

/CONTRAST (opleiding2niv)=Indicator

/CONTRAST (opleidingp2niv)=Indicator

/METHOD=ENTER groepnummer socsupbvprof stresservarenzw NABEVWERKENUREN weerwerknaxwkn opleiding2niv opleidingp2niv

/PLOT SURVIVAL HAZARDS

/PRINT=CI(95)

/CRITERIA=PIN(.05) POUT(.10) ITERATE(20).

COXREG cessexbfwk

/STATUS=cessexbf(1)

/PATTERN BY groepnummer

/CONTRAST (groepnummer)=Indicator

/METHOD=ENTER groepnummer

/PLOT SURVIVAL HAZARDS

/PRINT=CI(95)

/CRITERIA=PIN(.05) POUT(.10) ITERATE(20).

COXREG cessexbfwk

/STATUS=cessexbf(1)

/PATTERN BY groepnummer

/CONTRAST (groepnummer)=Indicator

/CONTRAST (ASTMA)=Indicator

/CONTRAST (GEBLANDNLJANEE)=Indicator

/CONTRAST (primapari)=Indicator

/CONTRAST (opleiding2niv)=Indicator

/CONTRAST (opleidingp2niv)=Indicator

/METHOD=ENTER groepnummer socsupbvprof stresservarenzw NABEVWERKENUREN weerwerknaxwkn opleiding2niv opleidingp2niv

/PLOT SURVIVAL HAZARDS

/PRINT=CI(95)

/CRITERIA=PIN(.05) POUT(.10) ITERATE(20).

* checken assumptie van proportionaliteit dmv log minus log plot > parallelle lijnen = ok*

DATASET ACTIVATE DataSet1.

COXREG cessbfwk

/STATUS=cessbf(1)

/STRATA=groepnummer

/PLOT LML

/CRITERIA=PIN(.05) POUT(.10) ITERATE(20).
